# Electrochemical Detection of Global DNA Methylation Using Biologically Assembled Polymer Beads

**DOI:** 10.3390/cancers13153787

**Published:** 2021-07-27

**Authors:** Narshone Soda, Zennia Jean Gonzaga, Amandeep Singh Pannu, Navid Kashaninejad, Richard Kline, Carlos Salomon, Nam-Trung Nguyen, Prashant Sonar, Bernd H. A. Rehm, Muhammad J. A. Shiddiky

**Affiliations:** 1 School of Environment and Science (ESC), Griffith University, Nathan Campus, Nathan, QLD 4111, Australia; n.soda@griffith.edu.au; 2 Queensland Micro- and Nanotechnology Centre (QMNC), Griffith University, Nathan Campus, Nathan, QLD 4111, Australia; n.kashaninejad@griffith.edu.au (N.K.); nam-trung.nguyen@griffith.edu.au (N.-T.N.); 3 Centre for Cell Factories and Biopolymers (CCFB), Griffith Institute for Drug Discovery (GRIDD), Griffith University, Nathan, QLD 4111, Australia; jean.gonzaga@griffithuni.edu.au; 4 Centre for Material Science, School of Chemistry and Physics, Queensland University of Technology, Brisbane, QLD 4001, Australia; a.pannu@qut.edu.au (A.S.P.); sonar.prashant@qut.edu.au (P.S.); 5 Centre for Biomedical Technologies, School of Chemistry and Physics, Queensland University of Technology (QUT), Brisbane, QLD 4001, Australia; 6 Division of Gynecologic Oncology, Department of Obstetrics and Gynecology, Ochsner Clinic Foundation, New Orleans, LA 70121, USA; rkline@ochsner.org; 7 Maternal-Fetal Medicine, Department of Obstetrics and Gynecology, Ochsner Clinic Foundation, New Orleans, LA 70121, USA; c.salomongallo@uq.edu.au; 8 Exosome Biology Laboratory, Centre for Clinical Diagnostics, University of Queensland Centre for Clinical Research, Royal Brisbane and Women’s Hospital, The University of Queensland, Brisbane, QLD 4029, Australia; 9 Departamento de Investigación, Postgrado y Educación Continua (DIPEC), Facultad de Ciencias de la Salud, Universidad Pedro de Valdivia, Santiago 8320000, Chile; 10 Menzies Health Institute Queensland (MHIQ), Griffith University, Gold Coast Campus, Gold Coast, QLD 4222, Australia

**Keywords:** electrochemical detection, DNA methylation, ovarian cancer, polyhydroxybuytrate nanobeads

## Abstract

**Simple Summary:**

Genomic profiling of cancer-derived materials in circulation has become an alternative approach for tumour genotyping. The detection of tumour origin markers such as DNA methylation in bodily fluids enables cancer screening, early-stage diagnosis and evaluation of therapy response. The development of broad platform technologies that underpin many in vitro clinical diagnostic tests has brought about a paradigm shift in cancer management and diagnosis. This study developed a multifaceted technology platform based on bioengineered polymer nanobeads for efficient capture and electrochemical detection of DNA methylation in ovarian cancer patient samples. This could be a versatile diagnostic platform for detecting numerous disease biomarkers, thus allowing several disease diagnoses.

**Abstract:**

DNA methylation is a cell-type-specific epigenetic marker that is essential for transcriptional regulation, silencing of repetitive DNA and genomic imprinting. It is also responsible for the pathogenesis of many diseases, including cancers. Herein, we present a simple approach for quantifying global DNA methylation in ovarian cancer patient plasma samples based on a new class of biopolymer nanobeads. Our approach utilises the immune capture of target DNA and electrochemical quantification of global DNA methylation level within the targets in a three-step strategy that involves (i) initial preparation of target single-stranded DNA (ss-DNA) from the plasma of the patients’ samples, (ii) direct adsorption of polymer nanobeads on the surface of a bare screen-printed gold electrode (SPE-Au) followed by the immobilisation of 5-methylcytosine (5mC)-horseradish peroxidase (HRP) antibody, and (iii) immune capture of target ss-DNA onto the electrode-bound PHB/5mC-HRP antibody conjugates and their subsequent qualification using the hydrogen peroxide/horseradish peroxidase/hydroquinone (H_2_O_2_/HRP/HQ) redox cycling system. In the presence of methylated DNA, the enzymatically produced (in situ) metabolites, i.e., benzoquinone (BQ), binds irreversibly to cellular DNA resulting in the unstable formation of DNA adducts and induced oxidative DNA strand breakage. These events reduce the available BQ in the system to support the redox cycling process and sequel DNA saturation on the platform, subsequently causing high Coulombic repulsion between BQ and negatively charged nucleotide strands. Thus, the increase in methylation levels on the electrode surface is inversely proportional to the current response. The method could successfully detect as low as 5% methylation level. In addition, the assay showed good reproducibility (% RSD ≤ 5%) and specificity by analysing various levels of methylation in cell lines and plasma DNA samples from patients with ovarian cancer. We envision that our bioengineered polymer nanobeads with high surface modification versatility could be a useful alternative platform for the electrochemical detection of varying molecular biomarkers.

## 1. Introduction

Epigenetic regulation of gene expression is expedited by mechanisms such as DNA methylation, histone modification and nucleosome positioning along the DNA [1]. The interaction between epigenetic elements enables a balance between transcription and repression by altering the chromatin architecture [2]. Thus, regulating DNA clusters ensures proper maintenance of precise chromosome replication, gene expression, and stable gene silencing [3]. DNA methylation is one of the most commonly occurring epigenetic events in the mammalian genome that plays a critical role in normal cell physiology [4]. It is a covalent chemical modification that results in adding a methyl group (-CH_3_) at the 5th position of the cytosine moiety observed within the cytosine-phosphate-guanine (CpG) dinucleotides. DNA methylation is essential for several cellular regulatory pathways such as X-chromosome inactivation, genomic imprinting, long term gene silencing and regulation of chromatin structure [5]. While appropriate DNA methylation is essential for normal biological processes, distinct and aberrant methylation patterns can result in many diseases, such as autoimmune diseases, diabetes, and cancer [2,6]. In the context of cancer, increasing studies have demonstrated that methylation of the promoter regions of several genes, including known tumour suppressor genes, results in the subsequent failure to express their functional proteins. Thus, aberrant DNA methylation patterns are a promising biomarker for early detection and assessment of future cancer risk owing to their early manifestation in carcinogenesis [7].

To date, various approaches for detecting global DNA methylation have been developed, including polymerase chain reaction (PCR) [8], combined bisulphite restriction analysis [9], enzyme-linked immunosorbent assay (ELISA) [10], high-performance liquid chromatography (HPLC)-based assay [11], high-performance capillary electrophoresis [12] and electrochemical approaches [13,14]. These conventional analysis approaches can provide superior information about the position and molar fraction of 5mC for each cytosine (C) within the DNA sequence. Among these approaches, electrochemical methods offer sensitive, simple, rapid, and low-cost analysis of global DNA methylation and are highly amenable to miniaturisation and multiplexing. Existing electrochemical global methylation assays mostly rely on the enzymatic reaction and amplification, electroactive species and DNA nucleobases affinity interactions to detect the overall DNA methylation. However, these techniques require long analysis intervals, experienced personnel, labels and expensive instrumentation, and can produce false-positive results, often occurring during the bisulphite conversion step [15]. These limitations hinder their widespread applicability in clinical settings. As such, precise quantification of methylation levels in both regional- and whole-genome provides a better understanding of cancer prognosis, diagnosis and aids the development of efficient therapies.

Epithelial ovarian cancer is one of the most prevalent gynaecological cancers in women and is commonly found in postmenopausal women after months of abdominal pain and distention. Advanced stage at diagnosis, poor prognosis and high incidence of resistance to therapy account for the most critical hurdles for ovarian cancer patients. Recently, our group has developed an amplification-free platform for the naked-eye observation and electrochemical quantification of long non-coding RNAs in ovarian cancer patients [16]. DNA methylation is another biomarker that plays a vital role in ovarian cancer, with several tumour suppressor genes shown to be hypermethylated [17]. Several studies have indicated that promoter hypermethylation is a universal mechanism for silencing tumor suppressor genes in human cancers, and it is estimated to be as common as mutation [17,18]. Recently, more attention has been focused on the methylation of *BRCA1* promoters since *BRCA1* mutations are known to be involved in inherited ovarian cancer [19]. Studies have also demonstrated *RASSF1A* promotes hypermethylation in ovarian cancer and might be one of the most frequently methylated genes in ovarian cancer [20]. Using DNA methylation as cancer-biomarkers may be exemplified in cancer treatment because they are chemically stable and cancer-associated changes in methylation predominantly precede tumour growth. Currently, ovarian cancer screening relies on the prevailing usage of blood-based CA125 protein biomarkers and transvaginal ultrasound, which can detect ovarian cancer in the preclinical phase in a substantial portion of cases. However, other factors can also result in elevated CA125 levels, such as menstruation, endometriosis, or ovarian cysts. As such, the lack of accurate disease risk classification during ovarian cancer screening has led to several health burdens associated with unnecessary biopsies and overtreatment of patients.

The continuous progress in the synthesis and processing of innovative nanomaterials that can be applied in bioassays has ushered into an era of nano-sized lab-on-chip technologies to detect disease-specific biomolecules. In particular, engineered nanomaterials such as polyhydroxybutyrate (PHB) nanobeads have recently been used in biosensing assays due to their unique properties, which can significantly improve the analytical specificity and sensitivity. Synthesised from *E. coli*, bioengineered PHB nanobeads represent a diverse family of biopolymers with unique thermal properties and mechanical characteristics useful for biosensing [21,22]. The biological assembly of PHB nanobeads enables the oriented immobilisation of various biomolecules on their surface for target-specific recognition and capture. It also makes them suitable for biomedical applications without inducing undesired immune responses. PHB itself is well tolerated by mammalian systems [23,24,25], and this has led to its use in implants such as bone scaffolding and other medical settings due to their biocompatibility and elastomeric properties [24,26]. 

Several studies have highlighted that DNA methylation sites can be specifically recognised using a family of proteins that contain conserved methyl-CpG binding domains [27,28]. Thus, methyl-CpG binding domains may be applied as specific analytical tools to detect methylated DNA sequences as well as MTase activity. The affinity interactions between anti-5-methylcytosine (5mC) and methylated ssDNA can be utilised to develop methods that can simultaneously detect methylation patterns at different genes or different gene-specific methylation loci [29] by hybridising the ssDNA captured onto 5mC antibody with appropriate complementary probes. 

In this work, we developed and studied a simple and rapid assay to detect global DNA methylation using PHB nanobeads in DNA samples obtained from patients with ovarian cancer and benign ones as a control group. To achieve a more extensive interacting active area on the sensing surface, PHB nanobeads were directly adsorbed on the sensing platform to simplify our assay design by avoiding multi-step sensor fabrication processes that are usually associated with DNA hybridisation-based assays. PHB beads had been engineered to display IgG binding ZZ domains [30,31,32,33,34] in order to bind to specific 5mC antibody peroxidase conjugate, and the resulting antibody-coated beads were then tested for highly specific and efficient capture of methylated DNA on the modified electrode surface. In the presence of HRP and HQ, the electrode generated enhanced amperometric responses via the H_2_O_2_/HRP/HQ redox cycling system. In the presence of methylated DNA, the enzymatically produced (in situ) metabolites, i.e., benzoquinone (BQ), binds irreversibly to cellular DNA resulting in the unstable formation of DNA adducts and induced oxidative DNA strand breakage [35,36,37]. These events reduce the available BQ metabolite in the system. In addition, the induced oxidative strand breaks result in the DNA saturation on the electrode surface. Under these circumstances, the Coulombic repulsion between BQ and negatively charged nucleotide strands repels additional BQ molecules to approach the electrode surface and therefore generates a reduced level of Faradaic current. This decreasing trend of Faradaic current response is inversely related to the amount of adsorbed methylated DNA, thus allowing for quantitative DNA methylation detection. 

## 2. Materials and Methods 

### 2.1. Reagents and Chemicals

All chemicals and reagents were of analytical grade and obtained from Sigma Aldrich (Sydney, NSW, Australia). UltraPure water was purchased from Invitrogen (Carlsbad, CA, USA). SPE-Au with a three-electrode system was purchased from Dropsens (Llanera, Spain). In the three-electrode system, working (diameter = 4 mm) and counter electrodes were gold, and the reference electrode was silver, respectively. Hydroquinone and hydrogen peroxide solution were purchased from Thermo Fisher Scientific Australia Pty Ltd. (Scoresby, VIC, Australia). 5-methylcytosine (5mC) antibody and horseradish peroxidase (HRP) conjugation kit were purchased from Abcam (Melbourne, VIC, Australia).

### 2.2. DNA Preparation

CpGenome human methylated and non-methylated DNA standard set Jurkat DNA (100% methylated) was purchased from Merck (Sydney, NSW, Australia). Whole-genome amplification (WGA) DNA was generated using the protocol of REPLI-g whole genome amplification kit (Qiagen Melbourne, VIC, Australia).

### 2.3. Preparation of DNA from Cell Line and Ovarian Cancer Samples 

Ovarian cancer cell line samples (SKOV3 and OVCAR3) together with a normal cell line (MeT-5A) were cultured using RPMI-1640 growth medium (Life Technologies, Australia) supplemented with 10% foetal bovine serum (Life Technologies, Melbourne, VIC, Australia) and 1% penicillin/streptomycin (Life Technologies, Melbourne, VIC, Australia). This was carried out in a humidified incubator with 5% CO_2_ flow at 37 °C. All cells were collected after four days for DNA extraction. For plasma sample collection, all subjects gave their informed consent for inclusion before they participated in the study. The study was conducted in accordance with the Declaration of Helsinki, and the protocol was approved by the Ethics Committee of the University of Queensland (approval number 2016000300) and the Ochsner Medical Center (New Orleans, LA, USA). Centrifugation at 2000× *g* for 10 min was performed to isolate plasma from the whole blood sample and subsequently stored at −80 °C until analysis. Appropriate procedures were used to collect the ovarian cancer samples and they were classified according to their histotype (e.g., stage I and stage III), and stored at −80 °C in the Biobank units. Only patients with epithelial ovarian cancer high-grade serous subtype (*n* = 6; P1–P6) and benign samples (*n* = 2; B1–B2) were used in this study. DNeasy Blood & Tissue Kits (Qiagen, Australia) was employed to extract DNA and the concentration was measured using a SPECTROstar Nano Microplate Reader (BMG Labtech, Melbourne, VIC, Australia) operated by MARS data analysis software. 

### 2.4. Electrochemical Detection of Methylated DNA Sequences

Electrochemical measurements were carried out using a CH1040C potentiostat (CH Instruments, Bee Cave, TX, USA) on screen-printed electrodes. Z_6_-PHB nanobeads were directly adsorbed on a bare SPE-Au surface followed by 5mC-HRP coupled antibody. Then, after denaturation at 95 °C, isolated genomic DNA was immobilised onto the surface-bound 5mC/HRP-PHB nanobead. The peroxidase activity of HRP via the H_2_O_2_/HRP/HQ redox cycling system was then used to achieve an electrochemical quantification of methylated DNA sequences present in the cell line and plasma solutions. The amperometric responses recorded throughout the manuscript correspond to the difference between the steady-state and the background currents and are an average of at least three replicates.

### 2.5. Preparation and Characterisation of PHB Nanobeads

*E. coli* BL21 (DE3) harbouring pMCS69 plasmid was transformed with pET14b_PHB (plain PHB nanobead negative control) and pET14b_Z_6_-PHB (PHB nanobeads displaying ZZ domains as positive control). The pMCS69 plasmid comprises the genes encoding PhaA and PhaB enzymes of *Ralstonia eutropha* that enables the production of the precursor *R*-3-hydroxybutyrate-coenzyme A mediating PHB production [38]. The recombinant *E. coli* BL21 (DE3) strains were grown at 37 °C until the optical density at 600 nm (OD_600_) reached 0.5–0.8 and subsequently induced by the addition of 1 mM isopropyl-*β*-d-thiogalactopyranoside (IPTG) (Goldbio, St. Louis County, MO, USA). The culture was incubated further for 48 h at 25 °C. The cells were harvested and PHB nanobeads were isolated and purified as previously described [39]. PHB nanobeads were stored in 10 mM Tris-HCl (pH 7.5) with 20% ethanol.

Nile-red staining and fluorescence microscopy (FM) were used to detect and analyse the cells producing PHB nanobeads as previously described [40]. The morphology of the PHB nanobeads was observed using transmission electron microscopy (TEM) and scanning electron microscopy (SEM). The preparation of samples for TEM followed the procedures previously described [41]. Prior to SEM analyses and imaging, the PHB nanobeads were subjected to immobilisation, drying and sputter-coated with an ultrathin layer of platinum in an argon atmosphere to make them electronically conductive. The average particle size and zeta-potential of the purified PHB nanobeads were determined by dynamic light scattering (DLS) at room temperature, using Zetasizer Nano-ZS (Malvern Panalytical, Worcestershire, UK). The nanobeads were diluted prior to size measurement to avoid multiple scattering effects. All measurements were performed in triplicate. To analyse the PHB content of the whole-cells and purified PHB nanobeads, the lyophilised samples were processed and subjected to crotonic analysis as previously described [42]. 

### 2.6. Electrode Surface Characterisation

The surface morphology of all the electrodes was investigated using a scanning electron microscope (SEM) and an atomic force microscope (AFM). A TESCAN MIRA3 FEG-SEM with an in-beam detector was used for SEM analysis. The AFM images were obtained with a Bruker Icon PT in air. The surface composition of the samples was analysed by XPS. The XPS spectra were recorded using an AXIS Supra photoelectron spectrometer (Kratos Analytical, Manchester, UK) with an aluminium anode to produce X-rays [Al Kα (hV = 1486.7 eV)]. All the electrodes were grounded with carbon tape to prevent charging. Charge compensation was also used to record the wide and high-resolution data from each sample

### 2.7. Functionality of PHB Nanobeads Displaying ZZ Domains

An IgG-binding capacity assay was used to assess the functionality of Z_6_-PHB nanobeads as previously described [43]. PHB nanobeads of 40–60 mg wet weight were used and washed twice with 1× PBS buffer (pH 7.5) by pipetting. Samples were centrifuged at 6000× *g* for four minutes between each wash in order to remove the ethanol. All samples were re-suspended in 500 μL 1× PBS buffer, and 5 mg of human IgG (Sigma-Aldrich, St. Louis, MO, USA) was added to each sample and incubated at 25 °C for 30 min with agitation to allow IgG binding to occur. After incubation, the tubes were centrifuged at 6000× *g* for four minutes and the unbound fraction of IgG in supernatants were aliquoted and analysed. The sediment was washed three times with 1× PBS buffer by pipetting, subsequently followed by centrifugation at 6000× *g* for four minutes between each wash. Bound IgG from PHB nanobeads was eluted by re-suspending the sediment in 0.5 mL of 50 mM Glycine (pH 2.7) and incubated at room temperature for five minutes with agitation. Then, the samples were centrifuged at 16,200× *g* for four minutes, and the eluted IgG in the supernatant was neutralised by adding 10 μL of 1 M K_2_HPO_4_ and analysed. Protein concentrations were analysed using the Bradford assay.

## 3. Results and Discussion

**Figure 1:**
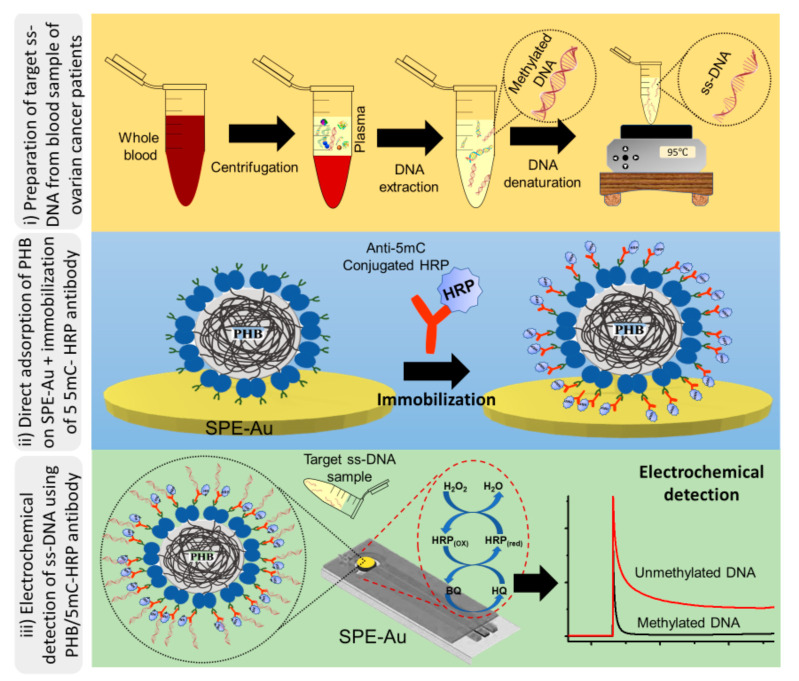
Schematic representation of DNA methylation assay using PHB nanobeads for modifying the electrode surface. Preparation of target ssDNA from blood samples of ovarian cancer patients (**top**) followed by direct immobilisation onto the surfaced bound 5mC/HRP-PHB complex. Direct adsorption of PHB onto SPE-Au and subsequent immobilisation of 5mC peroxidase conjugate (**middle**). The relative presence of methylated DNA is analysed by chronoamperometry in the presence of hydroquinone/H_2_O_2_ redox system (**bottom**).

**Figure 2:**
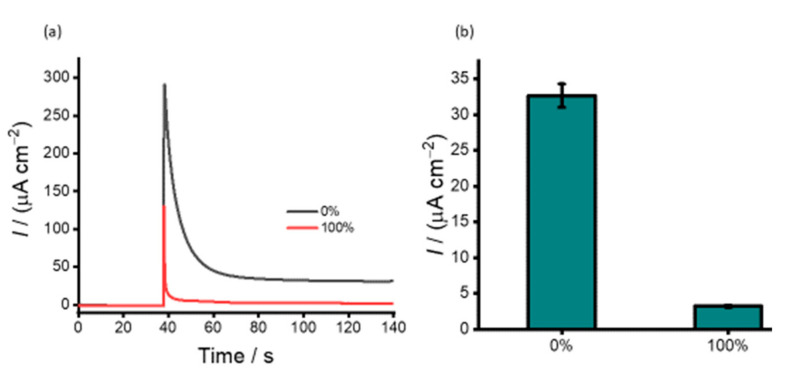
(**a**) Amperometric current density responses with respect to the presence (red) and absence (control) of 100% methylated synthetic sequences. (**b**) Corresponding current density profiles for the 100% target (right bar) and control (left bar) samples. Error bars represent the standard deviation of three repeat trials.

Figure 1 shows the electrochemical detection assay principle of DNA methylation. The first step in our assay preparation involves the direct adsorption of PHB nanobeads onto a bare SPE-Au surface. This was followed by immobilising a specific 5mC antibody for methylated DNA conjugated with HRP. Then, isolated genomic DNA was denatured to generate ssDNA, and immobilised onto the surface-bound 5mC/HRP-PHB nanobead. The peroxidase activity of HRP via the H_2_O_2_/HRP/HQ redox cycling system was then used to achieve an electrochemical quantification of methylated DNA sequences present in the cell line and plasma solutions. The functionality of the assay was determined by comparing the assay performance in detecting 100% of methylated DNA target sample. As observed in Figure 2, the total current density achieved with methylated DNA (100%, right bar) and WGA (0%, left bar) were 3.22 and 32.7 µA cm^−2^) respectively. The increased current response for WGA could be due to the HRP saturation level on the electrode surface, which allows a huge amount of available HQ to participate in the redox cycling processes. In the presence of methylated DNA (100%), a significant decrease in current response is observed. This may be attributed to the quinone metabolite generated in the redox cycling process, which facilitated the irreversible formation of unstable DNA adducts resulting in the deficiency of available metabolite in the redox cycling system. This may, therefore, steer a decrease in the current response. In addition, the oxidative DNA strand breaks induced by the electrophilic attack on DNA by BQ saturate the electrode surface with DNA, thereby increasing the Coulombic repulsion between BQ and negatively charged DNA [35,37]. The Coulombic repulsion of BQ ions away from the surface could significantly lower current for the 100% methylated sample compared to the unmethylated sample. This decrease in Faradaic current with respect to the baseline (0% methylation) is also inversely proportional to the amount of adsorbed methylated DNA, thus allowing for quantitative methylated DNA detection.

### 3.1. Characterisation of PHB Nanobeads

**Figure 3:**
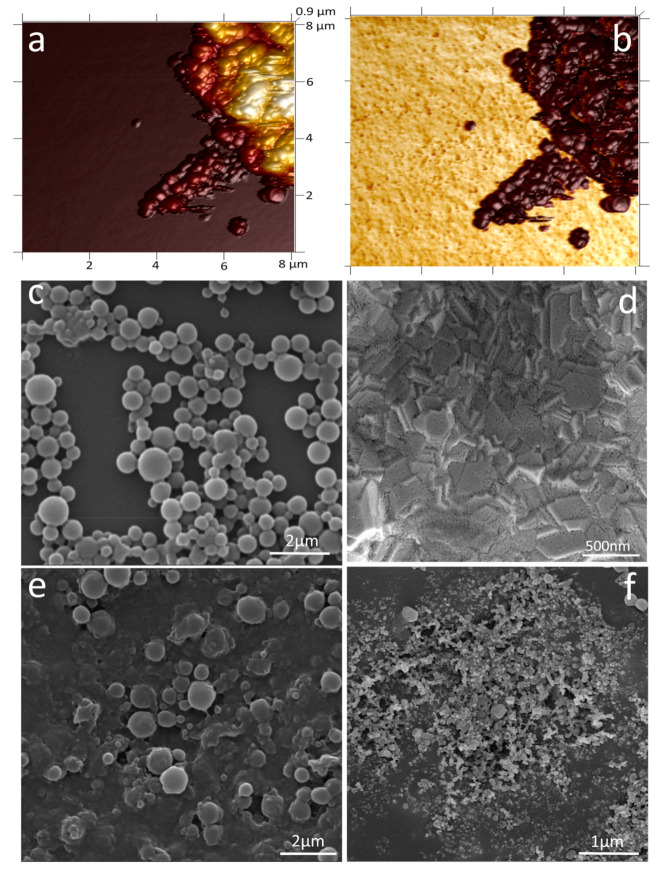
(**a**) AFM of PHB nanobeads on the gold electrode depicting height. (**b**) Adhesion profile of the same region differentiating the gold substrate (yellow) from the PHB beads (brown). SEM images of (**c**) PHB nanobeads, (**d**) bare SPE-Au electrode, (**e**) PHB-modified SPE-Au electrode, and (**f**) PHB-5mC/HRP modified SPE-Au electrode.

To confirm the direct adsorption of PHB nanobeads onto a bare SPE-Au surface, AFM of the electrode was recorded. The nominal height of PHB beads is depicted in Figure 3a, confirming the direct adsorption. The adhesion profile was studied (Figure 3b) to differentiate both PHB beads (dark brown) from the Au electrode surface (yellow). To investigate further the surface morphology of the electrode at each step, SEM of the electrode was recorded. Figure 3c, shows the spherical morphology of the purified PHB nanobeads, with Figure 3d depicting the surface of the SPE-Au electrode. Figure 2e confirms the adsorption of PHB beads on the Au electrode surface. The 5mC/HRP conjugate, immobilised onto the surface-bound PHB nanobeads, can be seen at two different scales in Figure 3f.

**Figure 4:**
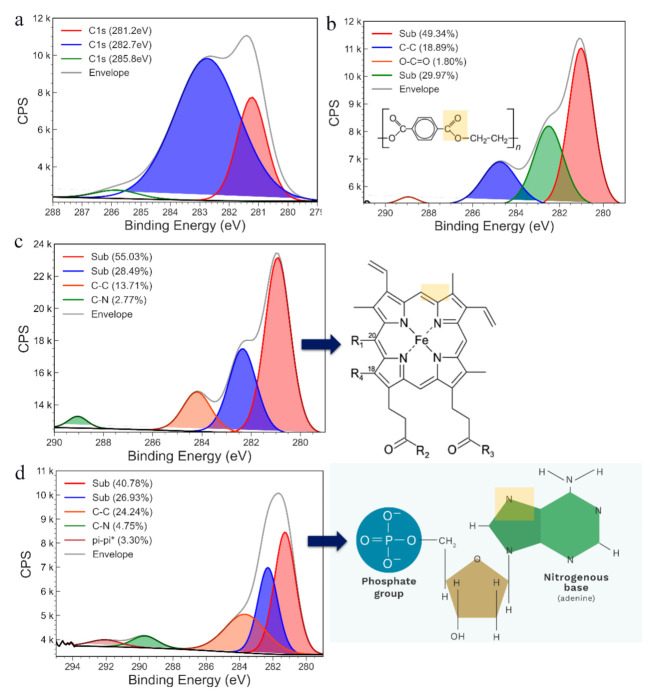
High-resolution C1s XPS spectrum of (**a**) bare SPE-Au electrode, (**b**) PHB-modified SPE-Au electrode, (**c**) PHB-5mC/HRP modified SPE-Au electrode, and (**d**) PHB-5mC/HRP modified SPE-Au electrode with ssDNA.

To further characterise the surface of the electrode after each modification step, XPS was recorded. The initial high-resolution spectrum of cleaned bare SPE-Au electrode indicates the presence of carbon on its surface (Figure 4a); hence a carbon element was chosen to monitor the further modification of the electrode. The C1s signal of the PHB-modified SPE-Au electrode (Figure 4b) shows the presence of C-C signal (18.89%) and O-C=O (1.8%), indicating chemical signal of PHB beads on the electrode surface. The rest of the signal can be attributed due to substrate interaction. The inset of Figure 4b shows the chemical structure of the PHB beads with highlights on bonds detected in the high-resolution C1s signal. The immobilisation of the 5mC peroxidase conjugate on the PHB-modified SPE-Au electrode was further verified by recording C1s high-resolution signal (Figure 4c) from the electrode. The presence of C–C bonds (13.71%) and C–N (2.77%) bonds, apart from the signal of the substrate, confirms bonding of peroxidase conjugate (inset of Figure 4c) onto the substrate. These modified electrodes were used in sensing ssDNA. After sensing, the C1s high-resolution signal (Figure 4d) was again recorded in XPS to investigate the binding of ssDNA. The presence of π–π bonds (3.30%), apart from C–C (24.24%) and C–N bonds (4.75%) again indicates successful binding of ssDNA and hence changing the C1s signal. The inset of Figure 4d shows the ssDNA and highlights the bonds detected in the C1s high-resolution XPS signal.

### 3.2. DNA Methylation Analysis in Heterogeneous Samples

**Figure 5:**
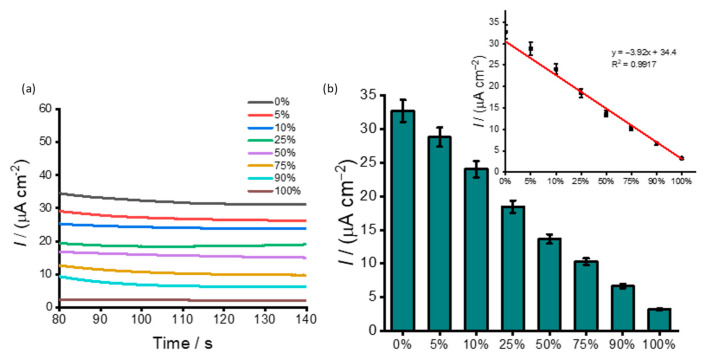
(**a**) Amperometric curves corresponding to different % methylated synthetic oligonucleotides sequences in buffer solution (i.e., samples containing 0%, 5%, 10%, 25%, 50%, 75%, 90%, and 100%); (**b**) their corresponding plots showing the magnitude current density profiles. Each data point represents the average of three repetitive experiments, and error bars represent the standard deviation of measurements (%RSD ≤ 5% for *n* = 3).

Global DNA methylation plays a major role in the pathogenesis of various types of genes by altering their expression [44,45,46]. As such, precise detection of global DNA methylation level in complex biological specimens could aid in diagnosing and prognosis of human cancer [47]. Recent reports have indicated that the presence of heterogeneous methylation in biological specimens often leads to difficulties in accurate detection and quantification of methylation with most samples consisting of both healthy and diseased cells [48]. Henceforth, it is imperative to quantify the heterogeneous methylation in a juxtaposed background. To assess the ability of the assay to distinguish heterogeneous DNA methylation, synthetic samples were designed by mixing 100% methylated Jurkat and 0% methylated WGA in different volume ratios to generate DNA samples containing different proportions (%) of methylation (i.e., 0%, 5% 10%, 50%, 75%, 90%, and 100%). These samples were then directly adsorbed on the modified SPE-Au surface under optimised conditions and analysed via electrochemical readout methods (Figure 5). The current density was decreasing with increasing methylation level. Fully methylated Jurkat samples exhibited the lowest current density, while the unmethylated WGA samples resulted in the highest current density. This can be explained by the fact that (i) lowering the amount of available HQ in the redox cycling process and (ii) increasing the Coulombic repulsion between the BQ and surface-attached DNA adducts increase with increasing the methylated levels in the target DNA sequences. As outlined above in Section 3.1, this combined effect significantly reduces the Faradaic current with increasing levels of methylation (Figure 5). The linear regression equation was found to be y = −3.92x + 34.4 (C) with a correlation coefficient (R^2^) of 0.9917. A methylation change as low as 5% could be detected. This data can be accredited to PHB nanobeads’ superior physicochemical properties that provide a huge active surface area for antibody and target immobilisation. The relative standard deviation of three independently fabricated sensors for detecting 10 ng uL^−1^ methylated target DNA was <5%, indicating acceptable precision and fabrication reproducibility. Comparable results have also been reported previously based on colourimetry [49,50] and gold–DNA [13,51] affinity interactions-based approach. Our previous study also reported a similar methylation change as low as 5% [52]. In this method, superparamagnetic PHB nanobeads exhibited specific binding to 5mC antibody, and superparamagnetic properties enabled magnetic separation of methylated DNA and subsequent detection via coupled peroxidase-mediated electrochemical reactions. Additionally, our approach is highly comparable to various conventional methods such as HPLC [53] and mass spectrometry methods [54]. However, these methods evaluate all cytosines across the genome to quantify methylation, whereas our method only screens a subset of methylated CpG sites for quantification of global methylation. The lowest detectable methylation change and reproducibility of the assay over a wide range of methylation levels with low input DNA samples demonstrate the potential applicability of PHB nanobeads as electrode surface modifiers for enhanced sensitive electrochemical signal. 

### 3.3. Detection of DNA Methylation in Cell Line Samples

**Figure 6:**
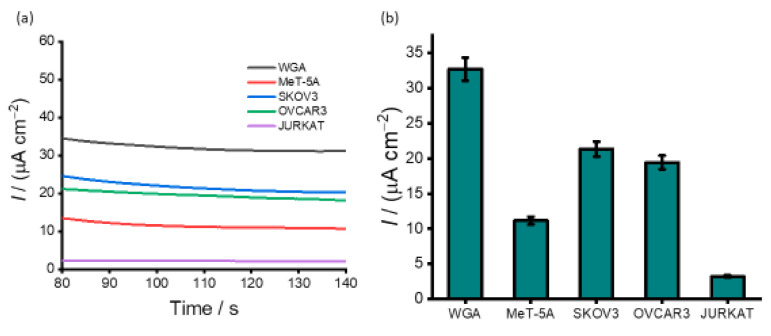
Analysis of ovarian cancer cell lines and a normal cell line. (**a**) Amperometric response for positive ovarian cancer cell line (SKOV3 and OVCAR3), normal cell line (MeT-5A) and internal controls (WGA and Jurkat); (**b**) corresponding current density profiles. Each data point represents the average of three repetitive trails, and error bars represent the standard deviation of measurements (%RSD ≤ 5%, for *n* = 3).

Metastasis, accounting for about 90% of cancer-related fatalities, remains a persistent challenge in cancer research [55]. Effective treatment or prevention of metastasis is intricate due to the heterogeneous nature of tumour which complicates therapeutic interventions. Consequently, understanding the tumour microenvironment is crucial to decipher the factors influencing metastasis and treatment responses. Ovarian cancer, frequently diagnosed at advanced stages with existing metastasis, offers a distinctive and valuable opportunity for studying the tumour microenvironment. To test the applicability of our assay for detecting methylation levels in complex biological samples, DNA samples derived from two ovarian cancer cell lines (SKOV 3 and OVCAR 3) and one non-cancerous cell line (MeT-5A) were tested (Figure 6). A fully unmethylated whole genome amplified (WGA) DNA and fully methylated Jurkat were used as internal standards. As anticipated, for all the cell lines and WGA samples, a substantial current density response was observed indicating the presence of different statuses of methylation. Similar to the synthetic DNA experiments, the relative current density response for SKOV3 and OVCAR3 was significantly lower (21.3 and 19.4 µA cm^−2^) compared to WGA (32.7 µA cm^−2^), indicating that DNA sequences derived from SKOV3 and OVCAR3 could be hypermethylated at the promoter gene. The chronoamperometric analysis shows that the current density changes derived from the cell lines are easily detectable against that of the control. (Figure 6b). This result is in agreement with our previously reported methylation levels in ovarian cancer cell lines [13]. The methylation level of the non-cancerous cell line MeT-5A (11.2 µA cm^−2^) is much lower than that of SKOV3 and OVCAR3, indicating hypomethylation at the promoter gene. These results demonstrate that SKOV3 exhibits over 35% methylation and OVCAR3 more than 41%. The cell line data shows good reproducibility of our assay (% RSD of <4.25% for *n* = 3) for the inter-assay signals for analysing DNA methylation levels in the ovarian cancer cell line without prior amplification or pre-treatment. The methylation statuses obtained for the cell lines indicate that the proposed assay may be an alternative for detecting global methylation in cell-derived samples.

### 3.4. Analysis of DNA Methylation in Clinical Samples

**Figure 7:**
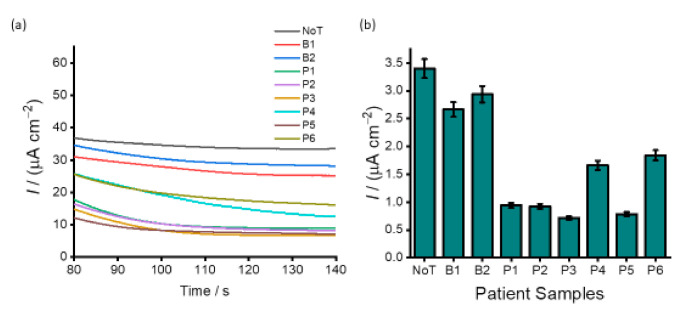
Analysis of patient samples. (**a**) Current density responses obtained for two benign ovarian cancer patients (B1-B2) and six patients of high-grade serous subtype ovarian cancer (P1–P5); (**b**) corresponding bar figure showing the magnitude of the current density response obtained for benign and high-grade serous subtype ovarian patient samples. Each data point represents the average of three repeat experiments, and error bars represent the standard deviation of measurements (%RSD ≤ 5% for *n* = 3).

The applicability of our assay to analyse clinical samples was demonstrated by analysing six plasma DNA samples (P1–P6) derived from patients with epithelial ovarian cancer. Two ovarian benign tissue DNA samples (B1 and B2) were also used as a control. As depicted in Figure 7 all samples exhibited various methylation levels. The current density response for NoT with respect to that of the benign samples clearly show the partial methylation for the two samples. More so, by juxtaposing the current density responses obtained in cell lines (Figure 6), we can estimate that six DNA samples derived from patients with high-grade serous ovarian cancer were highly methylated. The developed assay provides several benefits, such as efficient target recognition. In this case, PHB nanobeads were processed to exhibit specific binding to 5mC antibody via the Fc domain, and this enabled specific and efficient recognition of methylated DNA sequences. The direct binding of target methylated DNA sequences onto the PHB modified surface reduces the assay time by eliminating several time-consuming modifications and functionalisation steps usually involved in conventional assays. In addition, our assay relies on the use of disposable and cost-effective screen-printed electrodes and can detect DNA methylation in complex biological samples without prior PCR amplification and sequencing analysis.

## 4. Conclusions

This study describes the development of a simple and new method for quantifying DNA methylation events using PHB nanobeads. The method illustrates the application of PHB nanobeads as electrode surface modifiers, which facilitate the adsorption of a specific antibody for methylated DNA. The detection was achieved by direct immobilisation of various DNA samples onto a 5mC/HRP-PHB nanobead modified electrode. This method eliminates several modifications and functionalisation steps involved in conventional assays. In addition, it also avoids PCR amplification and the need for sequencing analysis. Most importantly, we have demonstrated the clinical feasibility of our method to detect methylation levels in ovarian cell lines and clinical samples from ovarian cancer patients. Unlike conventional methods, our assay’s high analytical efficiency, ease of use and low sample input may be useful for routine clinical diagnostics and a variety of other applications. It is worth emphasising that our assay design was to measure the samples’ total DNA methylation content (global DNA methylation) and not methylation of the CpG island of a specific gene. However, the assay can be modified to target gene-specific methylation.

Considering the great importance of DNA methylation in biological processes, our assay may present an important step towards developing a biosensor that could be useful for detecting disease-specific methylated DNA in bodily fluids (e.g., blood, urine, and saliva). Given that PHB nanobeads have high surface modification versatility for efficient capture and detection of different targets, we believe that the proposed assay could also be a multifaceted diagnostic platform for detecting a myriad of disease biomarkers, thus enabling multiple diseases diagnoses.

## Data Availability

Data is contained within the article or are available from the corresponding author upon reasonable request.

## References

[1] Jones P.A. (2012). Functions of DNA methylation: Islands, start sites, gene bodies and beyond. Nat. Rev. Genet..

[2] Robertson K.D. (2005). DNA methylation and human disease. Nat. Rev. Genet..

[3] Esteller M. (2007). Cancer epigenomics: DNA methylomes and histone-modification maps. Nat. Rev. Genet..

[4] Bird A. (2002). DNA methylation patterns and epigenetic memory. Genes Dev..

[5] Robertson K.D., Jones P.A. (2000). DNA methylation: Past, present and future directions. Carcinogenesis.

[6] Kulis M., Esteller M. (2010). DNA methylation and cancer. Adv. Genet..

[7] Laird P.W. (2003). The power and the promise of DNA methylation markers. Nat. Rev. Cancer.

[8] Yang A.S., Estécio M.R., Doshi K., Kondo Y., Tajara E.H., Issa J.P.J. (2004). A simple method for estimating global DNA methylation using bisulfite PCR of repetitive DNA elements. Nucleic Acids Res..

[9] Xiong Z., Laird P.W. (1997). COBRA: A sensitive and quantitative DNA methylation assay. Nucleic Acids Res..

[10] Kremer D., Metzger S., Kolb-Bachofen V. (2012). Quantitative measurement of genome-wide DNA methylation by a reliable and cost-efficient enzyme-linked immunosorbent assay technique. Anal. Biochem..

[11] Armstrong K.M., Bermingham E.N., Bassett S.A., Treloar B.P., Roy N.C., Barnett M.P. (2011). Global DNA methylation measurement by HPLC using low amounts of DNA. Biotechnol. J..

[12] Stach D., Schmitz O.J., Stilgenbauer S., Benner A., Döhner H., Wiessler M., Lyko F. (2003). Capillary electrophoretic analysis of genomic DNA methylation levels. Nucleic Acids Res..

[13] Bhattacharjee R., Moriam S., Nguyen N.-T., Shiddiky M.J. (2019). A bisulfite treatment and PCR-free global DNA methylation detection method using electrochemical enzymatic signal engagement. Biosens. Bioelectron..

[14] Hossain T., Mahmudunnabi R.G., Masud M.K., Islam N., Ooi L., Konstantinov K., Hossain S., Martinac B., Alici G., Nguyen N.-T. (2017). Electrochemical biosensing strategies for DNA methylation analysis. Biosens. Bioelectron..

[15] Laird P.W. (2010). Principles and challenges of genome-wide DNA methylation analysis. Nat. Rev. Genet..

[16] Soda N., Umer M., Kashaninejad N., Kasetsirikul S., Kline R., Salomon C., Nguyen N.-T., Shiddiky M.J.A. (2020). PCR-free detection of long non-coding HOTAIR RNA in ovarian cancer cell lines and plasma samples. Cancers.

[17] Hentze J.L., Høgdall C., Høgdall E.V. (2019). Methylation and ovarian cancer: Can DNA methylation be of diagnostic use? (Review). Mol. Clin. Oncol..

[18] Jones P.A., Baylin S.B. (2002). The fundamental role of epigenetic events in cancer. Nat. Rev. Genet..

[19] Baldwin R.L., Nemeth E., Tran H., Shvartsman H., Cass I., Narod S., Karlan B.Y. (2000). BRCA1 promoter region hypermethylation in ovarian carcinoma: A population-based study. Cancer Res..

[20] Wu Q., Lothe R.A., Ahlquist T., Silins I., Tropé C.G., Micci F., Nesland J.M., Suo Z., Lind G.E. (2007). DNA methylation profiling of ovarian carcinomas and their in vitro models identifies HOXA_9_, HOXB_5_, SCGB_3_A_1_, and CRABP_1_ as novel targets. Mol. Cancer.

[21] Rehm B., Steinbüchel A. (1999). Biochemical and genetic analysis of PHA synthases and other proteins required for PHA synthesis. Int. J. Biol. Macromol..

[22] Moradali M.F., Rehm B.H.A. (2020). Bacterial biopolymers: From pathogenesis to advanced materials. Nat. Rev. Genet..

[23] Chung C.W., Kim H.W., Kim Y.B., Rhee Y.H. (2003). Poly (ethylene glycol)-grafted poly (3-hydroxyundecenoate) networks for enhanced blood compatibility. Int. J. Biol. Macromol..

[24] Hazer B., Steinbüchel A. (2007). Increased diversification of polyhydroxyalkanoates by modification reactions for industrial and medical applications. Appl. Microbiol. Biotechnol..

[25] Legat A., Gruber C., Zangger K., Wanner G., Stan-Lotter H. (2010). Identification of polyhydroxyalkanoates in *Halococcus* and other haloarchaeal species. Appl. Microbiol. Biotechnol..

[26] Parlane N.A., Gupta S., Rubio-Reyes P., Chen S., Gonzalez-Miro M., Wedlock N., Rehm B.H.A. (2016). Self-assembled protein-coated polyhydroxyalkanoate beads: Properties and biomedical applications. ACS Biomater. Sci. Eng..

[27] Lewis J.D., Meehan R., Henzel W., Maurer-Fogy I., Jeppesen P., Klein F., Bird A. (1992). Purification, sequence, and cellular localization of a novel chromosomal protein that binds to methylated DNA. Cell.

[28] Meehan R., Lewis J.D., McKay S., Kleiner E.L., Bird A. (1989). Identification of a mammalian protein that binds specifically to DNA containing methylated CpGs. Cell.

[29] Xu Y., Gao X., Zhang L., Chen D., Dai Z., Zou X. (2016). Simultaneous detection of double gene-specific methylation loci based on hairpin probes tagged with electrochemical quantum dots barcodes. J. Electroanal. Chem..

[30] Ogura K., Rehm B.H.A. (2019). Alginate encapsulation of bioengineered protein-coated polyhydroxybutyrate particles: A new platform for multifunctional composite materials. Adv. Funct. Mater..

[31] Grage K., McDermott P., Rehm B.H.A. (2017). Engineering *Bacillus megaterium* for production of functional intracellular materials. Microb. Cell Factories.

[32] Lewis J.G., Rehm B.H. (2009). ZZ polyester beads: An efficient and simple method for purifying IgG from mouse hybridoma supernatants. J. Immunol. Methods.

[33] Mifune J., Grage K., Rehm B.H.A. (2009). Production of functionalized biopolyester granules by recombinant *Lactococcus lactis*. Appl. Environ. Microbiol..

[34] Brockelbank J.A., Peters V., Rehm B.H.A. (2006). Recombinant *Escherichia coli* strain produces a ZZ domain displaying biopolyester granules suitable for immunoglobulin G purification. Appl. Environ. Microbiol..

[35] Schlosser M.J., Shurina R.D., Kalf G.F. (1990). Prostaglandin H synthase catalyzed oxidation of hydroquinone to a sulfhydryl-binding and DNA-damaging metabolite. Chem. Res. Toxicol..

[36] Puckett-Vaughn D.L., Stenken J.A., Scott D., Lunte S.M., Lunte C.E. (1993). Enzymatic formation and electrochemical characterization of multiply substituted glutathione conjugates of hydroquinone. Life Sci..

[37] Lévay G., Ross D., Bodell W.J., György L. (1993). Peroxidase activation of hydroquinone results in the formation of DNA adducts in HL-60 cells, mouse bone marrow macrophages and human bone marrow. Carcinogenesis.

[38] Amara A.A., Rehm B.H.A. (2003). Replacement of the catalytic nucleophile cysteine-296 by serine in class II polyhydroxyalkanoate synthase from *Pseudomonas aeruginosa*-mediated synthesis of a new polyester: Identification of catalytic residues. Biochem. J..

[39] Reyes P.R., Parlane N.A., Wedlock N., Rehm B.H. (2016). Immunogencity of antigens from *Mycobacterium tuberculosis* self-assembled as particulate vaccines. Int. J. Med. Microbiol..

[40] Spiekermann P., Rehm B.H.A., Kalscheuer R., Baumeister D., Steinbüchel A.J.A.O.M. (1999). A sensitive, viable colony staining method using Nile red for direct screening of bacteria that accumulate polyhydroxyalkanoic acids and other lipid storage compounds. Arch. Microbiol..

[41] Evert B., Vezina B., Rehm B.H.A. (2020). Catalytically active bioseparation resin utilizing a covalent intermediate for tagless protein purification. ACS Appl. Bio Mater..

[42] Lemgruber R.D.S.P., Valgepea K., Tappel R., Behrendorff J.B., Palfreyman R.W., Plan M., Hodson M.P., Simpson S.D., Nielsen L.K., Köpke M. (2019). Systems-level engineering and characterisation of *Clostridium autoethanogenum* through heterologous production of poly-3-hydroxybutyrate (PHB). Metab. Eng..

[43] Jahns A.C., Maspolim Y., Chen S., Guthrie J.M., Blackwell L.F., Rehm B.H.A. (2013). In vivo self-assembly of fluorescent protein microparticles displaying specific binding domains. Bioconjug. Chem..

[44] Hao J.-J., Lin D.-C., Dinh H., Mayakonda A., Jiang Y.-Y., Chang C., Jiang Y., Lu C.-C., Shi Z.-Z., Xu X. (2016). Spatial intratumoral heterogeneity and temporal clonal evolution in esophageal squamous cell carcinoma. Nat. Genet..

[45] Mazor T., Pankov A., Song J., Costello J.F. (2016). Intratumoral heterogeneity of the epigenome. Cancer Cell.

[46] Bhattacharjee R., Moriam S., Umer M., Nguyen N.-T., Shiddiky M.J.A. (2018). DNA methylation detection: Recent developments in bisulfite free electrochemical and optical approaches. Analyst.

[47] Mikeska T., Candiloro I.L.M., Dobrovic A. (2010). The implications of heterogeneous DNA methylation for the accurate quantification of methylation. Epigenomics.

[48] Candiloro I.L., Mikeska T., Dobrovic A. (2011). Assessing combined methylation–sensitive high-resolution melting and pyrosequencing for the analysis of heterogeneous DNA methylation. Epigenetics.

[49] Wee E.J.H., Ngo T.H., Trau M. (2015). Colorimetric detection of both total genomic and loci-specific DNA methylation from limited DNA inputs. Clin. Epigenetics.

[50] Haque H., Bhattacharjee R., Islam N., Gopalan V., Nguyen N.-T., Lam A.K., Shiddiky M.J.A. (2017). Colorimetric and electrochemical quantification of global DNA methylation using a methyl cytosine-specific antibody. Analyst.

[51] Ibn Sina A.A., Howell S., Carrascosa L.G., Rauf S., Shiddiky M.J.A., Trau M. (2014). eMethylsorb: Electrochemical quantification of DNA methylation at CpG resolution using DNA–gold affinity interactions. Chem. Commun..

[52] Soda N., Gonzaga Z.J., Chen S., Koo K.M., Nguyen N.-T., Shiddiky M.J.A., Rehm B.H.A. (2021). Bioengineered polymer nanobeads for isolation and electrochemical detection of cancer biomarkers. ACS Appl. Mater. Interfaces.

[53] Kuo K.C., McCune R.A., Gehrke C.W., Midgett R., Ehrlich M. (1980). Quantitative reversed-phase high performance liquid chromatographic determination of major and modified deoxyribonucleosides in DNA. Nucleic Acids Res..

[54] Singer J., Schnute W.C., Shively J.E., Todd C.W., Riggs A.D. (1979). Sensitive detection of 5-methylcytosine and quantitation of the 5-methylcytosine/cytosine ratio in DNA by gas chromatography-mass spectrometry using multiple specific ion monitoring. Anal. Biochem..

[55] Jiménez Sánchez A. (2019). Characterisation of the Tumour Microenvironment in Ovarian Cancer. Ph.D. Thesis.

