# Correction: Soda et al. Electrochemical Detection of Global DNA Methylation Using Biologically Assembled Polymer Beads. *Cancers* 2021, *13*, 3787

**DOI:** 10.3390/cancers16071438

**Published:** 2024-04-08

**Authors:** Narshone Soda, Zennia Jean Gonzaga, Amandeep Singh Pannu, Navid Kashaninejad, Richard Kline, Carlos Salomon, Nam-Trung Nguyen, Prashant Sonar, Bernd H. A. Rehm, Muhammad J. A. Shiddiky

**Affiliations:** 1 School of Environment and Science (ESC), Griffith University, Nathan Campus, Nathan, QLD 4111, Australia; n.soda@griffith.edu.au; 2 Queensland Micro- and Nanotechnology Centre (QMNC), Griffith University, Nathan Campus, Nathan, QLD 4111, Australia; n.kashaninejad@griffith.edu.au (N.K.); nam-trung.nguyen@griffith.edu.au (N.-T.N.); 3 Centre for Cell Factories and Biopolymers (CCFB), Griffith Institute for Drug Discovery (GRIDD), Griffith University, Nathan, QLD 4111, Australia; jean.gonzaga@griffithuni.edu.au; 4 Centre for Material Science, School of Chemistry and Physics, Queensland University of Technology, Brisbane, QLD 4001, Australia; a.pannu@qut.edu.au (A.S.P.); sonar.prashant@qut.edu.au (P.S.); 5 Centre for Biomedical Technologies, School of Chemistry and Physics, Queensland University of Technology (QUT), Brisbane, QLD 4001, Australia; 6 Division of Gynecologic Oncology, Department of Obstetrics and Gynecology, Ochsner Clinic Foundation, New Orleans, LA 70121, USA; rkline@ochsner.org; 7 Maternal-Fetal Medicine, Department of Obstetrics and Gynecology, Ochsner Clinic Foundation, New Orleans, LA 70121, USA; c.salomongallo@uq.edu.au; 8 Exosome Biology Laboratory, Centre for Clinical Diagnostics, University of Queensland Centre for Clinical Research, Royal Brisbane and Women’s Hospital, The University of Queensland, Brisbane, QLD 4029, Australia; 9 Departamento de Investigación, Postgrado y Educación Continua (DIPEC), Facultad de Ciencias de la Salud, Universidad Pedro de Valdivia, Santiago 8320000, Chile; 10 Menzies Health Institute Queensland (MHIQ), Griffith University, Gold Coast Campus, Gold Coast, QLD 4222, Australia

In the original publication [1], **reference** [55] was not cited. The citation has now been inserted in **3. Results and Discussion, 3.3. Detection of DNA Methylation in Cell Line Samples**, and should read:

“Metastasis, accounting for about 90% of cancer-related fatalities, remains a persistent challenge in cancer research [55]. Effective treatment or prevention of metastasis is intricate due to the heterogeneous nature of tumour which complicates therapeutic interventions. Consequently, understanding the tumour microenvironment is crucial to decipher the factors influencing metastasis and treatment responses. Ovarian cancer, frequently diagnosed at advanced stages with existing metastasis, offers a distinctive and valuable opportunity for studying the tumour microenvironment. To test the applicability of our assay for detecting methylation levels in complex biological samples, DNA samples derived from two ovarian cancer cell lines (SKOV 3 and OVCAR 3) and one non-cancerous cell line (MeT-5A) were tested (Figure 6). A fully unmethylated whole genome amplified (WGA) DNA and fully methylated Jurkat were used as internal standards. As anticipated, for all the cell lines and WGA samples, a substantial current density response was observed indicating the presence of different statuses of methylation. Similar to the synthetic DNA experiments, the relative current density response for SKOV3 and OVCAR3 was significantly lower (21.3 and 19.4 µA cm^−2^) compared to WGA (32.7 µA cm^−2^), indicating that DNA sequences derived from SKOV3 and OVCAR3 could be hypermethylated at the promoter gene. The chronoamperometric analysis shows that the current density changes derived from the cell lines are easily detectable against that of the control. (Figure 6b). This result is in agreement with our previously reported methylation levels in ovarian cancer cell lines [13]. The methylation level of the non-cancerous cell line MeT-5A (11.2 µA cm^−2^) is much lower than that of SKOV3 and OVCAR3, indicating hypomethylation at the promoter gene. These results demonstrate that SKOV3 exhibits over 35% methylation and OVCAR3 more than 41%. The cell line data shows good reproducibility of our assay (% RSD of <4.25% for *n* = 3) for the inter-assay signals for analysing DNA methylation levels in the ovarian cancer cell line without prior amplification or pre-treatment. The methylation statuses obtained for the cell lines indicate that the proposed assay may be an alternative for detecting global methylation in cell-derived samples.”

The newly added reference appear below:

55. Jiménez Sánchez, A. Characterisation of the Tumour Microenvironment in Ovarian Cancer. Ph.D. Thesis, University of Cambridge, Cambridge, UK, 2019. https://doi.org/10.17863/CAM.35250.

With this correction, the order of some references has been adjusted accordingly. The authors apologize for any inconvenience caused and state that the scientific conclusions are unaffected. This correction was approved by the Academic Editor. The original publication has also been updated.

## References

[1] Soda N., Gonzaga Z.J., Pannu A.S., Kashaninejad N., Kline R., Salomon C., Nguyen N.T., Rehm B.H.A., Shiddiky M.J.A. (2021). Electrochemical Detection of Global DNA Methylation Using Biologically Assembled Polymer Beads. Cancers.

